# Effect of mineralocorticoid receptor antagonists on cardiac function in patients with heart failure and preserved ejection fraction: a systematic review and meta-analysis of randomized controlled trials

**DOI:** 10.1007/s10741-018-9758-0

**Published:** 2019-01-07

**Authors:** Chris J. Kapelios, Jonathan R. Murrow, Thomas G. Nührenberg, Maria N. Montoro Lopez

**Affiliations:** 1 Department of Health Policy, The London School of Economics and Political Science, London, UK; 2 Cardiology Department, Laiko General Hospital, 17 Agiou Thoma Street, 11 527 Athens, Greece; 3 Augusta University—University of Georgia Medical Partnership, Athens, GA USA; 4 Department of Cardiology and Angiology II, University Heart Center Freiburg • Bad Krozingen, Bad Krozingen, Germany; 5 Heart Failure Department, Heart Hospital, Hamad Medical Corporation, Doha, Qatar

**Keywords:** Mineralocorticoid receptor antagonist, Spironolactone, Eplerenone, Heart failure, Diastolic function

## Abstract

Heart failure with preserved ejection fraction (HFpEF) is a disease with limited evidence-based treatment options. Mineralocorticoid receptor antagonists (MRA) offer benefit in heart failure with reduced ejection fraction (HFrEF), but their impact in HFpEF remains unclear. We therefore evaluated the effect of MRA on echocardiographic, functional, and systemic parameters in patients with HFpEF by a systematic review and meta-analysis. We searched MEDLINE, EMBASE, clinicaltrials.gov, and Cochrane Clinical Trial Collection to identify randomized controlled trials that (a) compared MRA versus placebo/control in patients with HFpEF and (b) reported echocardiographic, functional, and/or systemic parameters relevant to HFpEF. Studies were excluded if: they enrolled asymptomatic patients; patients with HFrEF; patients after an acute coronary event; compared MRA to another active comparator; or reported a follow-up of less than 6 months. Primary outcomes were changes in echocardiographic parameters. Secondary end-points were changes in functional capacity, quality of life measures, and systemic parameters. Quantitative analysis was performed by generating forest plots and calculating effect sizes by random-effect models. Between-study heterogeneity was assessed through *Q* and *I*^2^ statistics. Nine trials with 1164 patients were included. MRA significantly decreased E/e′ (mean difference − 1.37, 95% confidence interval − 1.72 to − 1.02), E/A (− 0.04, − 0.08 to 0.00), left ventricular end-diastolic diameter (− 0.78 mm, − 1.34 to − 0.22), left atrial volume index (− 1.12 ml/m^2^, − 1.91 to − 0.33), 6-min walk test distance (− 11.56 m, − 21 to − 2.13), systolic (− 4.75 mmHg, − 8.94 to − 0.56) and diastolic blood pressure (− 2.91 mmHg, − 4.15 to − 1.67), and increased levels of serum potassium (0.23 mmol/L, 0.19 to 0.28) when compared with placebo/control. In patients with HFpEF, MRA treatment significantly improves indices of cardiac structure and function, suggesting a decrease in left ventricular filling pressure and reverse cardiac remodeling. MRA increase serum potassium and decrease blood pressure; however, a small decrease in 6-min-walk distance is also noted. Larger prospective studies are warranted to provide definitive answers on the effect of MRA in patients with HFpEF.

**Electronic supplementary material:**

The online version of this article (10.1007/s10741-018-9758-0) contains supplementary material, which is available to authorized users.

## Introduction

Heart failure (HF) with preserved ejection fraction (HFpEF) is a clinical entity for which few evidence-based treatments exist [1]. Mineralocorticoid receptor antagonists (MRA), which represent a cornerstone treatment for patients with HF with reduced ejection fraction (HFrEF), failed to decrease mortality in HFpEF patients in a large, multi-center trial [2]. Nevertheless, the significant criticism pertaining to the trial’s design and execution [3, 4], combined with the paucity of other relevant, large data have resulted in uncertainty regarding the use of MRA in HFpEF. A small number of well-performed randomized studies have reported positive results with the use of MRA in HFpEF when assessing cardiac function by echocardiographic or patients’ functional status [5–9].

Still, the most recent European Society of Cardiology Guidelines state that “evidence that MRA improve symptoms in these patients is lacking”, while no reference is made to the reported effects of MRA on cardiac function and structure, as well as other systemic parameters [1]. On the other hand, US guidelines take a more definitive, though guarded stance (IIB recommendation) [10], in view of the positive impact of MRA observed on ventricular remodeling [11], favorable adaptations in echocardiographic parameters of diastolic function, and subgroup analysis of a secondary hospitalization endpoint from a clinical trial [12, 13]. Given that a significant body of literature has been published since the last relevant meta-analysis of randomized trials [14–18], a new meta-analysis on the topic seems timely and warranted. Inclusion of data on functional and systemic parameters, apart from echocardiographic indices, would further strengthen the rationale for performing the meta-analysis.

The objective of the current study was to critically assess the effect of MRA on echocardiographic, functional, and systemic parameters in adult patients with HFpEF included in randomized controlled trials.

## Methods

The present study was conducted according to the PRISMA statement (Table A1-Appendix) [19]. The review protocol has been registered in PROSPERO: International Prospective Register of Systematic Reviews (Number: CRD42018104929) www.crd.york.ac.uk/PROSPERO/display_record.php?ID=CRD42018104929, Accessed 18 August 2018.

### Identification and selection of studies

MEDLINE, EMBASE, clinicaltrials.gov, and Cochrane Clinical Trial Collection were searched on June 15, 2018 with a combinatorial approach (Boolean operator “AND”) of three broader search terms. The broader search terms were derived using the Boolean operator “OR” between synonyms for “heart failure,” “preserved ejection fraction,” and “mineralocorticoid receptor antagonists.” Detailed descriptions of the terms used for MEDLINE and EMBASE searches are outlined in the Appendix Tables A2 and A3. The search was restricted to the period from January 1, 2000 onwards, out of concern for a high risk of imprecision in the clinical diagnosis of HFpEF prior to 2000. Only articles written in English were eligible, while there was no restriction regarding publication status. The reference lists from previous systematic reviews relevant to our topic were hand-screened for studies [11, 14, 20], whereas references of the included articles were screened for additional studies. If needed, authors were contacted to request unpublished original papers or further details not available on the official version.

Study eligibility criteria included: (a) comparison of MRA (spironolactone, eplerenone, canrenone) with placebo/control; (b) adult patients diagnosed with HFpEF; (c) follow-up ≥ 6 months, as administration of MRA for a shorter period was considered unlikely to produce significant functional and echocardiographic changes; and (d) report of the outcomes of interest (Table 1). HFpEF definition included patients with HF symptoms and left ventricular ejection fraction (LVEF) ≥ 45%. There was no cut-off for natriuretic peptides or cardiac abnormalities as these criteria have been recently established by guidelines and would have excluded most of the older trials. We excluded studies if they included patients with previous history of myocardial infarction or compared MRA with another active comparator. Ethical approval was not required, as no patients were recruited.

**Table 1:** Primary and secondary outcomes included in the analysis

Primary	Echocardiographic parameters (E/e′, E/A, deceleration time, LVEDD, LVEF, LAVi, LVMi)
Secondary	A. Functional parameters (VO_2_ peak, 6MWD, NYHA, QoL)
	B. Systemic parameters (SBP, DBP, natriuretic peptides, serum potassium)

*DBP* diastolic blood pressure, *LAVi* left atrial volume index, *LVMi* left ventricular mass index, *LVEDD* left ventricular end-diastolic diameter, *LVEF* left ventricular ejection fraction, *NYHA* New York Heart Association, *QoL* quality of life, *SBP* systolic blood pressure, *VO*_*2*_ oxygen consumption, *6MWD* 6-min walk test distance

The search was independently performed by two reviewers (MML and TN). End-note was used to remove duplicates. All titles and abstracts were screened individually by all four reviewers, in order to select those that met the inclusion criteria. Differences in assessment of eligibility between reviewers were resolved through discussion and consensus.

### Risk of bias

Risk of bias (RoB) within studies was assessed using the Cochrane Risk of Bias Tool. The assessment was performed at the study level and regarded components recommended by the Cochrane Collaboration for randomized trials, namely randomization sequence generation, treatment allocation concealment, blinding, completeness of outcome data, and selective outcome [21]. For each component, trials were categorized as low, high, or unclear risk of bias. Studies that were deemed to be at high risk of bias would only be included in the systematic review but not in the meta-analysis.

Risk of bias across studies was evaluated by assessing publication bias and selective reporting within studies. In order to explore publication bias (meta-bias), unpublished information was meticulously searched so that it could be incorporated to quantitative analysis. Α list of the conference databases that were searched to this end is given in Table A4 (Appendix). We assessed quality of evidence for outcomes using GRADE criteria [22].

In addition, protocol registries (clinicaltrials.gov and PROSPERO) were scanned to assess selective outcome reporting. All four reviewers performed their personal assessment and any disagreements were discussed until consensus was reached.

### Data extraction

A systematic approach was used to extract the relevant variables from the selected studies. The variables for which data were sought are shown in detail in Table A5 (Appendix) and regarded study identity and design, patient population, intervention, and outcomes. Outcome parameters were divided into three groups: echocardiographic, functional, and systemic parameters. Echocardiographic parameters were prioritized over the other parameters as primary outcomes, as they are systematically measured and reported in relevant studies. Furthermore, several echocardiographic indices (LAVi, E/e′) have been recognized as significant predictors of prognosis in patients with HFpEF [23, 24], thus entailing clinical implications to our analysis. Volumetric echocardiographic parameters that were not indexed were not included. Quality of life (QoL) changes should have been estimated with either Kansas City Questionnaire (KCCQ) or the Minneapolis Living With Heart Failure Questionnaire (MLWHFQ). All four reviewers extracted study characteristics and data input was cross-validated between reviewer databases.

### Qualitative and statistical analysis

Data were combined in a systematic review, forest plots and, if appropriate, in a meta-analysis. We set three studies as the minimum number for quantitative synthesis of data in a meta-analysis for each study parameter. Given that all outcomes were continuous variables, mean differences were used as effect measures. For those parameters, which were indexed to diverging bases across studies (i.e., left ventricular mass index, LVMI) or quantified by different measurement techniques (i.e., BNP and NT-proBNP), standardized mean differences by the method of Hedges were used. When not available, standard deviations were derived from confidence intervals according to the Cochrane Handbook for Systematic Reviews of Interventions or from median with interquartile ranges according to Wan et al. [21, 25]. Missing standard deviations for the difference between treatment and control group were also calculated according to the Cochrane Handbook for Systematic Reviews of Interventions [21].

Our data was expected to be heterogeneous due to the relatively small sample sizes and the diversity in study design. Consequently, analysis was performed with random effect models. Heterogeneity was assessed by the *Q* statistic; however, due to its limited power to rule out heterogeneity, a *p* value threshold of 0.10 was used. A quantitative analysis of the impact of heterogeneity using the *I*^2^ statistic was also performed. *I*^2^ values > 50% were considered as highly heterogenous. In order to evaluate the effect of imputing standard deviations of within-group changes, a sensitivity analysis for different values of correlation coefficients (0.7, 0.9, and 0.8 or calculated based on given study data) was performed.

Although performance of three subgroup analyses to explore heterogeneity had been pre-specified (multi-center vs single-center studies, studies with high percentage of women vs even gender distribution and studies with high vs low baseline use of diuretics), subgroup analysis was not considered feasible due to the small number of studies. For the same reason, we also decided not to perform meta-regression analyses and funnel plots with the trial mean differences to explore meta-bias. All *p* values were two-tailed with statistical significance set at 0.05 (if not otherwise specified) and confidence intervals (CI) computed at 95% level. All analyses were performed with the use of Stata 15 Software (StataCorp LLC, Texas, US).

## Results

### Identified and eligible studies

**Fig. 1:**
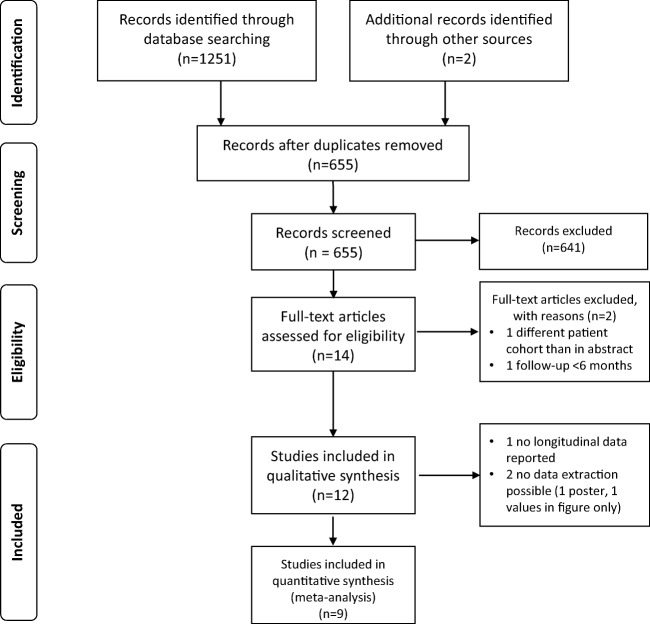
PRISMA flow chart of study identification and selection

The number of identified and screened studies is indicated in Fig. 1.

Our initial search identified 1253 studies from 2000 onwards; after removal of duplicates, screening of titles, abstracts, and full-texts and adding studies from previous analyses up to 2014, 12 studies were included in the qualitative synthesis (Table 2). Of these, three studies were not suitable for data extraction, which resulted in nine studies amenable for inclusion in the meta-analysis.

**Table 2:**
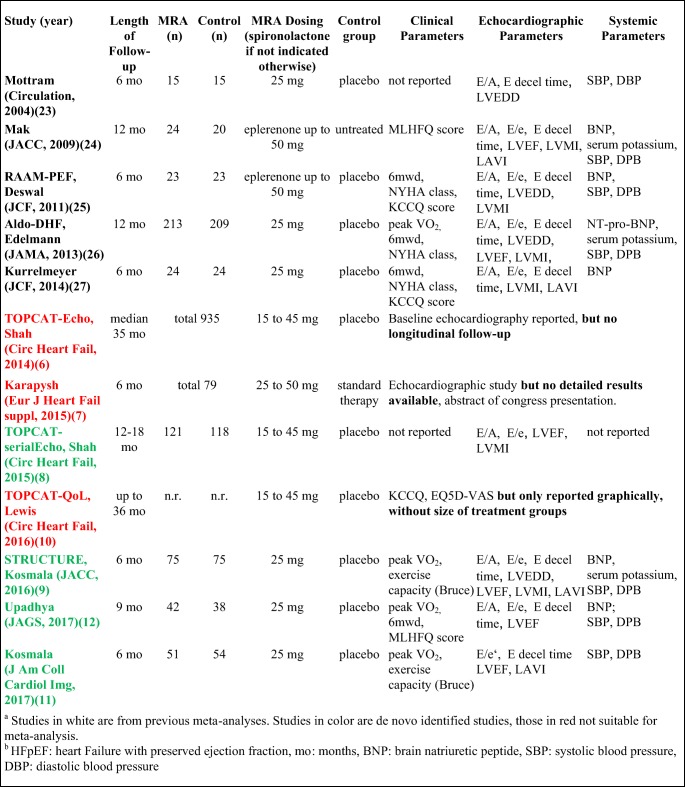
Characteristics of studies included in the qualitative analysis

Studies in white are from previous meta-analyses. Studies in color are de novo identified studies, those in red not suitable for meta-analysis

*HFpEF* heart failure with preserved ejection fraction, *mo* months, *BNP* brain natriuretic peptide, *SBP* systolic blood pressure, *DBP* diastolic blood pressure

### Characteristics of included studies

The studies enrolled 1164 patients (588 patients in the MRA and 576 in the control/placebo arm) who were followed for up to 18 months. Of the included trials, seven included patients with a left ventricular ejection fraction ≥ 50% and eight were placebo controlled. In seven studies, the administered MRA was spironolactone, while in the remaining two studies eplerenone. The baseline characteristics and the reported clinical, echocardiographic parameters and systemic parameters are indicated for each study in Table A6 (Appendix).

### Risk of bias within studies

**Fig. 2:**
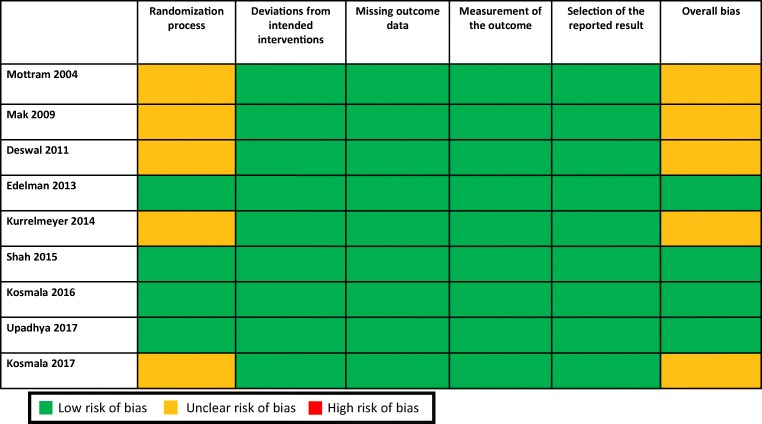
Risk of bias assessment for studies included in the meta-analysis

During quality assessment, issues regarding random sequence generation were observed in 5 studies. Protocol deviations, incomplete outcome data and selective reporting of results were not observed in any of the selected studies. Details of RoB assessment are given in Fig. 2.

### Risk of bias across studies

Multiple conference databases as well as protocol registries were scanned for other studies that could be relevant to our meta-analysis. Our search did not produce any results that could indicate any concerns regarding publication or selective outcome reporting biases.

### Results of individual studies and synthesis of results

#### Echocardiographic parameters

Echocardiographic parameters were frequently measured and reported among studies. Eight studies reported E/A and E/e′, while seven studies provided data on deceleration time (DT). Six studies included data on left ventricular mass index (LVMi) and ejection fraction (LVEF) and four on left ventricular end-diastolic diameter (LVEDD). Finally, values for left atrial volume index (LAVi) were provided by five studies.

**Fig. 3:**
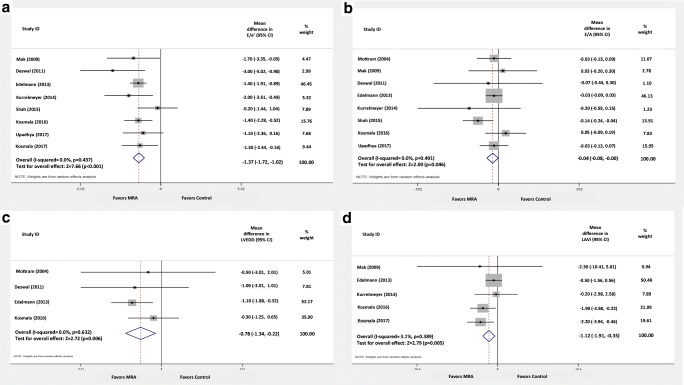
Mean difference estimates of **A** E/e′, **B** E/A, **C** left ventricular end-diastolic diameter (LVEDD), and **D** left atrial volume index (LAVi) of MRA versus control

MRA compared to placebo/control significantly decreased E/e′ (mean difference [MD]: − 1.37; 95% CI: − 1.02 to − 1.72; comparison *p* < 0.001; heterogeneity *p* = 0.437; *I*^2^ = 0.0%), E/A (MD: − 0.04; 95% CI: − 0.08 to 0.0; comparison *p* = 0.046; heterogeneity *p* = 0.491; *I*^2^ = 0.0%), LVEDD (MD: − 0.78 mm; 95% CI: − 1.34 to − 0.22; comparison *p* = 0.006; heterogeneity *p* = 0.632; *I*^2^ = 0.0%), and LAVi (MD: − 1.12 ml/m^2^; 95% CI: − 1.91 to − 0.33; comparison *p* = 0.005; heterogeneity *p* = 0.389; *I*^2^ = 3.1%) (Fig. 3).

On the contrary, MRA compared to placebo/control did not significantly affect DT (MD: − 8.38 ms; 95% CI: − 21.76 to 5.00; comparison *p* = 0.220; heterogeneity *p* < 0.001; *I*^2^ = 85.5%), LVEF (MD: 0.62%; 95% CI: − 0.65 to 1.88; comparison *p* = 0.340; heterogeneity *p* = 0.155; *I*^2^ = 37.7%), and LVMi (standardized MD: − 0.12; 95% CI: − 0.50 to 0.27; comparison *p* = 0.550; heterogeneity *p* < 0.001; *I*^2^ = 84.5%) (Appendix Fig. 1).

#### Functional parameters

Functional parameters were assessed and reported in less than 50% of included studies. Four studies reported maximum rate of oxygen consumption (VO_2_ peak), 6-min walk test distance (6-MWD), and New York Heart Association (NYHA) class. Quality of life (QoL) metrics were assessed and presented in five studies.

**Fig. 4:**
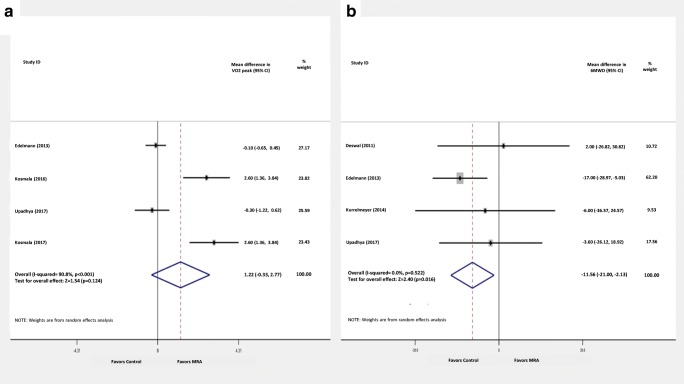
Mean difference estimates of **A** VO_2_ peak and **B** 6-min walk distance (6-MWD) of MRA versus control

MRA compared to placebo/control, did not significantly increase VO_2_ peak (MD: 1.22 ml/kg/min; 95% CI: − 0.33 to 2.77; comparison *p* = 0.124; heterogeneity *p* < 0.001; *I*^2^ = 90.8%), while they significantly decreased 6-MWD (MD: − 11.56 m; 95% CI: − 21 to − 2.1; comparison *p* = 0.016; heterogeneity *p* = 0.522; *I*^2^ = 0.0%) (Fig. 4).

Among other measures of functional status, NYHA class was treated as a categorical rather than a continuous variable in three out of four studies, rendering the pooling of effect estimates unfeasible. Regarding QoL measures, two studies used the KCCQ, in this way not fulfilling the pre-specified cut-off for quantitative synthesis. Three studies used the MLWHFQ; MRA use did not significantly affect MLWHFQ (MD: − 1.15; 95% CI: − 3.00 to 0.693; comparison *p* = 0.221; heterogeneity *p* = 0.597; *I*^2^ = 0.0%) (Appendix Fig. 2). Furthermore, as the two questionnaires have inverse directions for high-quality (MLWHFQ scale of 0–105 with higher scores indicating lower QoL and KCCQ scale of 0–100 with higher scores indicating higher QoL) pooling of estimates with standardized effect sizes was considered inappropriate.

#### Systemic parameters

Systolic and diastolic blood pressure were common outcomes among the included studies, as they were reported in seven of them. On the other hand, serum potassium and blood levels of natriuretic peptides (BNP/NT-proBNP) were uncommon outcomes, as they were measured in three and four studies, respectively.

Among HFpEF patients, MRA treatment compared to placebo/control significantly decreased systolic (MD: − 4.75 mmHg; 95% CI: − 8.94 to − 0.56; comparison *p* = 0.026; heterogeneity *p* = 0.001; *I*^2^ = 74.6%) and diastolic blood pressure (MD: − 2.91 mmHg; 95% CI: − 4.15 to − 1.67; comparison *p* < 0.001; heterogeneity *p* = 0.350; *I*^2^ = 10.4%) (Appendix Fig. 3). Additionally, MRA significantly increased serum levels of potassium (MD: 0.23 mmol/L; 95% CI: 0.19 to 0.28; comparison *p* < 0.001; heterogeneity *p* = 0.539; *I*^2^ = 0.0%), but did not affect blood levels of BNP/NT-proBNP (standardized MD: − 0.00; 95% CI: − 0.29 to 0.28; comparison *p* = 0.970; heterogeneity *p* = 0.015; *I*^2^ = 64.5%) compared with placebo/control (Appendix Fig. 4).

#### Additional analyses

##### Sensitivity analysis

The effects of MRA on echocardiographic, functional, and systemic parameters of patients with HFpEF seemed to be largely insensitive to different levels of correlation coefficient (0.7, 0.9, and 0.8 or calculated based on given study data) for imputation of standard deviations of within-group changes (Table A7).

## Discussion

The main findings of this meta-analysis on the effect of MRA in patients with HFpEF are: MRA (a) positively affect significant echocardiographic indices of cardiac structure and function, (b) slightly decrease 6-MWD, (c) increase levels of serum potassium, and (d) decrease blood pressure.

Our study adheres to PRISMA reporting guidelines, while our conclusions are based on evidence of moderate to high quality (GRADE). Moreover, we exclusively included randomized controlled trials of patients with symptomatic HFpEF and not patients with asymptomatic diastolic dysfunction, HFrEF or myocardial infarction, as previous studies on the topic had done [11, 14, 20]. This was crucial in obtaining a comparable patient sample and analyzing multiple outcomes with low level of heterogeneity. Additionally, as we screened studies up to June 2018, we included in our quantitative analysis four new, randomized studies, which contributed approximately 50% of the overall population and increased statistical power of analysis and significance of findings [15–18]. Furthermore, our study meticulously studied a range of echocardiographic, functional, and systemic parameters, thus providing the only, to date, comprehensive review of these effects of MRA in the HFpEF patient population.

### Effect of MRA on echocardiographic parameters

Our study provides high-quality evidence to support that MRA can exert significant, positive effects on diastolic cardiac function (E/e′) and structure (LVEDD, LAVi) of HFpEF patients. Beneficial effects of spironolactone on clinical endpoints (HF hospitalizations) in these patients have been previously reported [2]; however, failure of one landmark trial to demonstrate superiority of spironolactone regarding the prespecified primary outcome has resulted in low MRA use among real-world HFpEF patients [23]. Elevated E/e′ and left ventricular (LVEDD) and left atrial dimensions (LAVi) have all been recognized as predictors of adverse clinical outcome in this patient population [24–30]. Whether these indices represent markers of unchangeable, progressive disease or of potentially reversible pathophysiological mechanisms remains poorly elucidated; however, our current understanding of the natural process of the disease and the close correlation of these parameters with LV filling pressure suggests that the latter is the most probable scenario underlining the therapeutic potential of MRA [31]. Hence, the above echocardiographic parameters could be considered as surrogate markers of clinical outcomes and, until otherwise proven, therapeutic targets. Furthermore, decrease of LVEDD and LAVi also suggests that MRA induce reverse remodeling.

### Effect of MRA on functional parameters

MRA use did not confer significant improvements in QoL indices in the present meta-analysis, though the neutral results may have been driven by the small number of studies reporting QoL parameters, alongside the use of two different questionnaires. Changes in NYHA class, though clinically significant, were not reported as numerical variables in the included studies and thus could not be quantitatively synthesized. Finally, inconsistencies in the effect of MRA on functional capacity of HFpEF patients were reported. Exercise capacity, objectively assessed by peak VO_2_, was increased by an increment of 1.2 ml/kg/min, though the result was statistically insignificant. Nonetheless, evaluation of this effect is hindered by high heterogeneity. Conversely, MRA led to significant, though mild decrease (11.5 m) in 6MWD in these patients. This effect, which was largely driven by the results of a single study [8], is contradictory to the other, physiological and clinical, effects of MRA in patients with HFpEF. Thus, further investigations are warranted to confirm the effect of MRA on functional parameters.

### Effect of MRA on systemic parameters

MRA use results in significant increases in serum potassium levels. In particular, serum potassium increased in the intervention group (from weighted mean 4.20 ± 0.0 to 4.39 ± 0.03 mmol/L) but decreased in the control group (from weighted mean 4.21 ± 0.03 to 4.17 ± 0.06 mmol/L). These findings are of clinical importance as previous studies have demonstrated that lower levels of serum potassium are associated with adverse outcome [32, 33], while high-normal levels of potassium are accompanied by the most favorable prognosis in HF patients [34]. On the other hand, MRA treatment also leads to significant decreases in blood pressure. Systolic (weighted mean 136.5 ± 8.4 to 130.7 ± 7.5 mmHg for intervention vs. 136.2 ± 6.4 to 135.2 ± 5.5 mmHg for control) and diastolic blood pressure (weighted mean 77.5 ± 2.7 to 74.6 ± 3.0 mmHg for intervention vs. 77.9 ± 3.6 to 77.2 ± 4.4 mmHg for control) decreased in both groups; however, magnitude of decrease was greater in the intervention group. This effect, which has been previously reported [35], is encouraging as multiple studies support that treating hypertension is of high relevance in HFpEF [36, 37].

### Clinical significance and future perspective

As mentioned above, the only multi-center study to date investigating the effect of MRA on outcomes failed to meet its primary endpoint [2]. One must not, however, disregard the signals of efficacy observed with the use of spironolactone compared with placebo (reduction of HF hospitalizations by 17%), as well as the regional disparities that may have confounded the results [3, 4]. Particularly, patients enrolled on the basis of the hospitalization criterion were much younger, with fewer coexisting conditions and a lower risk profile compared with patients enrolled on the basis of elevated natriuretic peptides [3]. Moreover, the former also had a lower event rate, a finding which contradicts a large body of HF literature. The majority of patients from Russia and Georgia were enrolled in the hospitalization stratum. Furthermore, based on the blood analyses of 366 patients participating in the study who were reporting to take the drug at 12 months, canrenone (spironolactone’s metabolite) concentrations were undetectable in a significantly higher proportion of participants from Russia than from the United States and Canada (30% vs. 3%), strongly suggesting that the trial results obtained in Russia may not reflect the true therapeutic response to spironolactone [4]. Importantly, a post hoc analysis demonstrated that spironolactone seemed to benefit patients in the Americas but not those in Russia or Georgia [3].

Nonetheless, given that new trials aiming to evaluate the effect of MRA on hard clinical endpoints are not expected for several years [38], effect of MRA on other clinically relevant parameters, such as the ones reported herein, may have important implications in informing physicians’ decision to administer MRA in HFpEF patients. Due to the relatively small study populations and the limitations of the studies in the field, several issues pertaining to the effects of MRA in HFpEF remain unsettled. Namely, the question whether the lack of significant effect of MRA on some studied parameters is due to type II error needs to be clarified via future larger studies. This pertains in particular to parameters which are either of high clinical significance (QoL, NYHA, BNP, LVMi) or/and for which a trend is reported in the present study (DT, VO_2_peak). New studies will also be needed to confirm or reject the unexpected result of decrease in 6-MWD with use of MRA. This finding is paradoxical as exercise capacity has been shown to increase (not decrease) alongside the aforementioned changes in cardiac function/structure [39].

#### Limitations

Our study shares the same weaknesses with previous systematic reviews in the field. First, heterogeneity in the criteria employed to diagnose HFpEF and in the design of studies represent major limitations, as they may have resulted in heterogeneous patient populations. Sample size was relatively small; thus, type II error may explain some of the negative findings of the analysis. Furthermore, study outcomes were not consistently reported in all included trials. All these limitations may in part be responsible for significant heterogeneity observed among the pooled analyses for some outcomes. Moreover, this meta-analysis was not performed on a patient level but collected aggregate data from randomized studies with different designs. This fact precluded performance of subgroup analysis in specific subpopulation. Although most included studies enrolled patients based on the same ejection fraction threshold (≥ 50%), other distinct inclusion criteria varied across studies. Hence, extrapolation of results to the overall HFpEF population should be done with caution.

Despite these caveats, this meta-analysis may have significant therapeutic implications. In view of aggregate favorable effects with no sound evidence of adverse effects, MRA treatment should be considered as a treatment option for patients with HFpEF.

## Conclusions

In patients with HFpEF, MRA use leads to significant improvements in important indices of cardiac structure and function, potentially indicating a decrease in LV filling pressure and reverse cardiac remodeling. MRA significantly increase serum potassium, decrease blood pressure, but also decrease 6-MWD. Although this study represents the most comprehensive, to date review of MRA effects on echocardiographic, functional, and systemic parameters in HFpEF, larger prospective studies are warranted to provide definitive answers.

## Electronic supplementary material

ESM 1 (DOCX 11073 kb)
