# Supplementary material for: Effect of mineralocorticoid receptor antagonists on cardiac function in patients with heart failure and preserved ejection fraction: a systematic review and meta-analysis of randomized controlled trials
Source: Heart Fail Rev. 2019 Jan 7;24(3):367–77. doi: 10.1007/s10741-018-9758-0 (PMC6477010; doi:10.1007/s10741-018-9758-0)
Supplement: Supplementary file 1 — (DOCX 11073 kb) [file 10741_2018_9758_MOESM1_ESM.docx]

**APPENDIX**

**Table A1. PRISMA 2009 Checklist***

| **Section/topic** | **#** | **Checklist item** | **Reported on page #** |
| --- | --- | --- | --- |
| **TITLE** | | |  |
| Title | 1 | Identify the report as a systematic review, meta-analysis, or both. | 1 |
| **ABSTRACT** | | |  |
| Structured summary | 2 | Provide a structured summary including, as applicable: background; objectives; data sources; study eligibility criteria, participants, and interventions; study appraisal and synthesis methods; results; limitations; conclusions and implications of key findings; systematic review registration number. | 2 |
| **INTRODUCTION** | | |  |
| Rationale | 3 | Describe the rationale for the review in the context of what is already known. | 4 |
| Objectives | 4 | Provide an explicit statement of questions being addressed with reference to participants, interventions, comparisons, outcomes, and study design (PICOS). | 4 |
| **METHODS** | | |  |
| Protocol and registration | 5 | Indicate if a review protocol exists, if and where it can be accessed (e.g., Web address), and, if available, provide registration information including registration number. | 4 |
| Eligibility criteria | 6 | Specify study characteristics (e.g., PICOS, length of follow-up) and report characteristics (e.g., years considered, language, publication status) used as criteria for eligibility, giving rationale. | 5 |
| Information sources | 7 | Describe all information sources (e.g., databases with dates of coverage, contact with study authors to identify additional studies) in the search and date last searched. | 5 |
| Search | 8 | Present full electronic search strategy for at least one database, including any limits used, such that it could be repeated. | Appendix |
| Study selection | 9 | State the process for selecting studies (i.e., screening, eligibility, included in systematic review, and, if applicable, included in the meta-analysis). | 5 |
| Data collection process | 10 | Describe method of data extraction from reports (e.g., piloted forms, independently, in duplicate) and any processes for obtaining and confirming data from investigators. | 7 |
| Data items | 11 | List and define all variables for which data were sought (e.g., PICOS, funding sources) and any assumptions and simplifications made. | Appendix |
| Risk of bias in individual studies | 12 | Describe methods used for assessing risk of bias of individual studies (including specification of whether this was done at the study or outcome level), and how this information is to be used in any data synthesis. | 5,6 |
| Summary measures | 13 | State the principal summary measures (e.g., risk ratio, difference in means). | 7 |
| Synthesis of results | 14 | Describe the methods of handling data and combining results of studies, if done, including measures of consistency (e.g., I^2^) for each meta-analysis. | 6,7 |
| Section/topic | # | Checklist item | Reported on page # |
| Risk of bias across studies | 15 | Specify any assessment of risk of bias that may affect the cumulative evidence (e.g., publication bias, selective reporting within studies). | 6 |
| Additional analyses | 16 | Describe methods of additional analyses (e.g., sensitivity or subgroup analyses, meta-regression), if done, indicating which were pre-specified. | 7 |
| **RESULTS** | | |  |
| Study selection | 17 | Give numbers of studies screened, assessed for eligibility, and included in the review, with reasons for exclusions at each stage, ideally with a flow diagram. | 7,8 |
| Study characteristics | 18 | For each study, present characteristics for which data were extracted (e.g., study size, PICOS, follow-up period) and provide the citations. | Appendix |
| Risk of bias within studies | 19 | Present data on risk of bias of each study and, if available, any outcome level assessment (see item 12). | 8 |
| Results of individual studies | 20 | For all outcomes considered (benefits or harms), present, for each study: (a) simple summary data for each intervention group (b) effect estimates and confidence intervals, ideally with a forest plot. | 8-10, Appendix |
| Synthesis of results | 21 | Present results of each meta-analysis done, including confidence intervals and measures of consistency. | 8-10, Appendix |
| Risk of bias across studies | 22 | Present results of any assessment of risk of bias across studies (see Item 15). | 8 |
| Additional analysis | 23 | Give results of additional analyses, if done (e.g., sensitivity or subgroup analyses, meta-regression [see Item 16]). | 10 |
| **DISCUSSION** | | |  |
| Summary of evidence | 24 | Summarize the main findings including the strength of evidence for each main outcome; consider their relevance to key groups (e.g., healthcare providers, users, and policy makers). | 10-12 |
| Limitations | 25 | Discuss limitations at study and outcome level (e.g., risk of bias), and at review-level (e.g., incomplete retrieval of identified research, reporting bias). | 12,13 |
| Conclusions | 26 | Provide a general interpretation of the results in the context of other evidence, and implications for future research. | 13 |
| **FUNDING** | | |  |
| Funding | 27 | Describe sources of funding for the systematic review and other support (e.g., supply of data); role of funders for the systematic review. | 13 |

* **This checklist has been adapted for use from Table 1 in Moher D, et al:** Preferred reporting items for systematic reviews and meta-analyses: the PRISMA statement. PLoS medicine. 2009;6(7):e1000097.

**Table A2. Search strategy for MEDLINE-Ovid**

1. exp Heart Failure, Diastolic/

2. diastolic heart failure.tw.

3. heart failure with normal ejection fraction.tw.

4. heart failure with preserved ejection fraction.tw.

5. left ventricular diastolic failure.tw.

6. diastolic dysfunction.tw.

7. HFpEF.tw.

8. 1 or 2 or 3 or 4 or 5 or 6 or 7

9. exp Mineralocorticoid Receptor Antagonists/

10. aldosterone blocker.mp.

11. aldosterone receptor antagonist.mp.

12. aldosterone blockade.mp.

13. spironolactone.mp. or exp SPIRONOLACTONE/

14. aldactone.mp.

15. Eplerenone.mp.

16. inspra.mp.

17. canrenoic acid.mp.

18. canrenoate potassium.mp.

19. canrenone.mp. or CANRENONE/

20. aldactone.mp. or Spironolactone/

21. mineralocorticoid receptor.mp. or Receptors, Mineralocorticoid/

22. mineralocorticoid receptor.mp. or Receptors, Mineralocorticoid/ or aldosterone/ or Receptors, Mineralocorticoid/ or Eplerenone.mp.

23. 9 or 10 or 11 or 12 or 13 or 14 or 15 or 16 or 17 or 18 or 19 or 20 or 21 or 22

24. heart failure.mp.

25. exp Heart Failure/

26. 24 or 25

27. 8 and 23 and 26

**Table A3. Search strategy for EMBASE-Ovid**

1. aldosterone antagonist/exp or aldosterone antagonist.mp.

2. mineralocorticoid antagonist/exp or mineralocorticoid antagonist.mp.

3. aldosterone blockade.mp.

4. aldosterone receptor antagonist.mp.

5. spironolactone/exp or spironolactone.mp. [mp=title, abstract, heading word, drug trade name, original title, device manufacturer, drug manufacturer, device trade name, keyword, floating subheading word, candidate term word]

6. aldactone.mp. or aldactone/exp or aldactone.mp. [mp=title, abstract, heading word, drug trade name, original title, device manufacturer, drug manufacturer, device trade name, keyword, floating subheading word, candidate term word]

7. 'inspra'/exp or 'inspra'.mp. [mp=title, abstract, heading word, drug trade name, original title, device manufacturer, drug manufacturer, device trade name, keyword, floating subheading word, candidate term word]

8. 'canrenoic acid'/exp or 'canrenoic acid'.mp. [mp=title, abstract, heading word, drug trade name, original title, device manufacturer, drug manufacturer, device trade name, keyword, floating subheading word, candidate term word]

9. 'canrenoate potassium'/exp or 'canrenoate potassium'.mp. [mp=title, abstract, heading word, drug trade name, original title, device manufacturer, drug manufacturer, device trade name, keyword, floating subheading word, candidate term word]

10. 'canrenone'/exp or 'canrenone'.mp.

11. 'eplerenone'/exp or 'eplerenone'.mp.

12. 'aldactone'/exp or 'aldactone'.mp.

13. 1 or 2 or 3 or 4 or 5 or 6 or 7 or 8 or 9 or 10 or 11 or 12

14. 'diastolic heart failure'/exp or 'diastolic heart failure'.mp. [mp=title, abstract, heading word, drug trade name, original title, device manufacturer, drug manufacturer, device trade name, keyword, floating subheading word, candidate term word]

15. 'diastole'.mp.

16. 'diastolic dysfunction'.mp.

17. 'heart failure with preserved ejection fraction'.mp.

18. 'heart failure preserved ejection fraction'.mp.

19. 'preserved ejection fraction'.mp.

20. 'left ventricular diastolic failure'.mp.

21. 'preserved left ventricular ejection fraction'.mp.

22. 'heart failure with normal ejection fraction'.mp.

23. 'preserved left ventricular function'.mp.

24. 14 or 15 or 16 or 17 or 18 or 19 or 20 or 21 or 22 or 23

25. 13 and 24

26. 'randomized controlled trial'/exp or 'randomized controlled trial'.mp.

27. 'clinical trial'/exp or 'clinical trial'.mp.

28. 'random$'.mp.

29. 'placebo$'.mp.

30. 'double blind method'.mp.

31. 'single blind method'/exp or 'single blind method'.mp.

32. 'double-blind'.mp.

33. 'single-blind'.mp.

34. 26 or 27 or 28 or 29 or 30 or 31 or 32 or 33

35. 13 and 24 and 34

36. limit 35 to (human and english language)

**Table A4. Conference databases that were scanned for studies relevant to the meta-analysis**

| American College of Cardiology |
| --- |
| American Heart Association |
| European Society of Cardiology |
| European Heart Failure Association |
| American Heart Failure Association |

**Table A5. Study characteristics collected and included in the analysis**

| **Study ID** | First author, corresponding author, journal, publication year |
| --- | --- |
| **Study design** | Number of groups, number of patients per group |
| **Patient population** | Sex, age, somatometric parameters, prevalence of comorbidities, baseline systemic status, baseline echocardiographic and functional status, relative drug therapy |
| **Intervention** | Type of MRA (eplerenone, spironolactone, canreonate), dose of MRA, duration of MRA administration, duration of follow-up, time-points of outcomes assessment (in case of multiple time-points the longest time-point up to a maximum of 12 months will be included) |
| **Outcomes** | Primary: echocardiographic parameters (E/e′, E/A, DT, LVEDD, LVEF, LAVi, LVMi)  Secondary: a) functional (VO2 peak, 6MWD, NYHA, QoL) and b) systemic parameters (SBP, DBP, Natriuretic peptides, serum potassium) |

**Table A6. Characteristics of included studies^+^**

| Study  (reference) | Special clinical features | LVEF | Follow-up (months) | MRA (n) | Control (n) | MRA Dosing  (spironolactone if not indicated otherwise) | Systemic  Parameters | Echo  Parameters | Functional Parameters |
| --- | --- | --- | --- | --- | --- | --- | --- | --- | --- |
| Mottram (5) * | NYHA≥II | ≥50% | 6 | 15 | 15 | 25 mg | SBP, DBP | E/A, DT, LVEDD | - |
| Mak (6) | - | >45% | 12 | 24 | 20 | eplerenone up to 50 mg | SBP, DBP, BNP, sPot | E/A, E/e‘, DT, LVMi. LAVi | MLWHFQ |
| Deswal (7) * | NYHA II-III | ≥50% | 6 | 23 | 23 | eplerenone up to 50 mg | SBP, DBP, BNP, sPot | E/A, E/e‘, DT, LVEDD, LVMi. | NYHA, 6MWD, KCCQ |
| Edelmann (8) * | Age>50 years,  NYHA II-III | ≥50% | 12 | 213 | 209 | 25 mg | SBP, DBP, NT-pro-BNP, sPot | E/A, E/e‘, DT, LVEDD, LVEF, LVMi. LAVi | NYHA, 6-MWD, peak VO_2,_ MLHFQ |
| Kurrelmeyer (9) * | Women NYHA II-III | ≥50% | 3,6 | 24 | 24 | 25 mg | BNP | E/A, E/e‘, DT, LVMi, LAVi | NYHA, 6-MWD, KCCQ |
| Shah (11) * | - | ≥45% | 12-18 | 121 | 118 | 15-45 mg | - | E/A, E/e‘, LVEF, LVMi. | - |
| Kosmala (12) * | NYHA II-III | ≥50% | 6 | 75 | 75 | 25 mg | BNP, sPot | E/A, E/e‘, DT, LVEDD, LVEF, LVMi. LAVi | peak VO_2_ |
| Upadhya (13) * | Older age 71±1 years | ≥50% | 4,9 | 42 | 38 | 25 mg | SBP, DBP, BNP | E/A, E/e‘, DT, LVEF | 6-MWD, peak VO_2_, MLWHFQ |
| Kosmala (14) * | NYHA II-III | ≥50% | 6 | 51 | 54 | 25 mg | SBP, DBP, BNP | E/e‘, DT, LVEF, LAVi | NYHA, peak VO_2_ |
|  |  |  |  | 588 | 576 |  |  |  |  |

^+^ DBP: diastolic blood pressure, DT: deceleration time, KCCQ: Kansas City Cardiomyopathy Questionnaire, LAVi: left atrial volume index, LVEDD: left ventricular end-diastolic diameter, LVEF: left ventricular ejection fraction, LVMi: left ventricular mass index, MLHFQ: Milwaukee Living with Heart Failure Questionnaire, MRA: mineralocorticoid receptor antagonist, NYHA: New York Heart Association, SBP: systolic blood pressure, sPot: serum potassium, 6-MWD: 6-minute walk distance, * use of placebo

**Table A7. Sensitivity analysis of mean differences (or standardized mean differences) based on standard deviations calculated with correlation coefficient of 0.7, 0.9 and 0.8 or calculated from available studies**

* *DBP: diastolic blood pressure; DT: deceleration time; LAVi: left atrial volume index; LVMi: left ventricular mass index; LVEDD: left ventricular end-diastolic diameter; LVEF: left ventricular ejection fraction; MD: mean difference; SBP: systolic blood pressure; SMD: standardized mean difference; VO2: oxygen consumption; 6MWT: 6-minute walk test*

**A.**


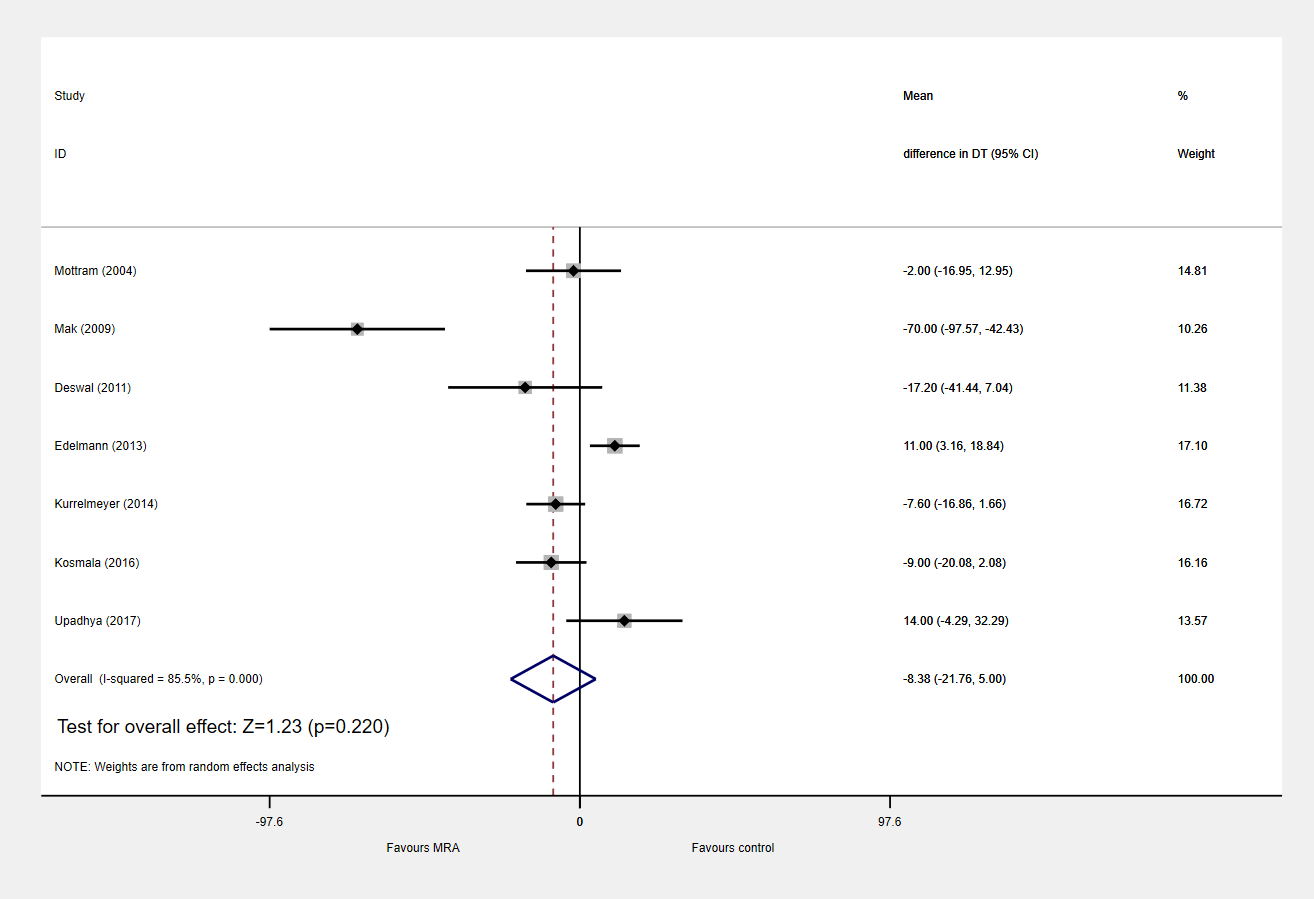


**B.**


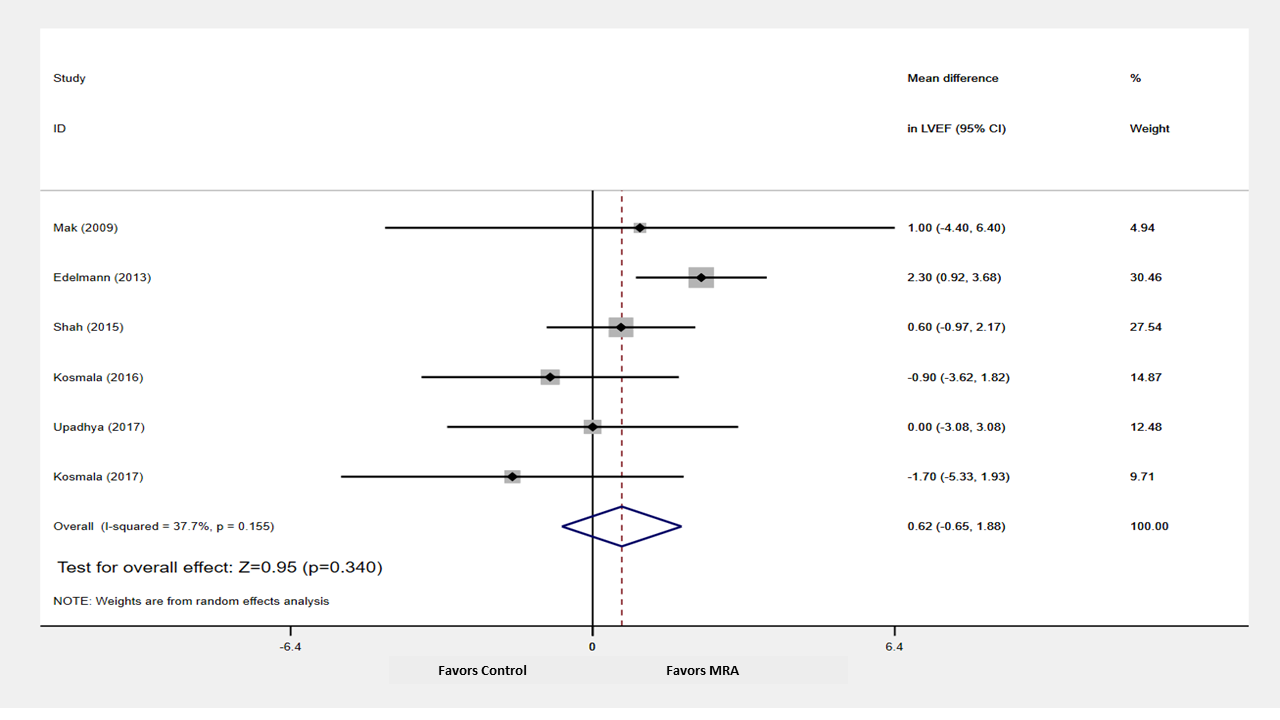


**C.**


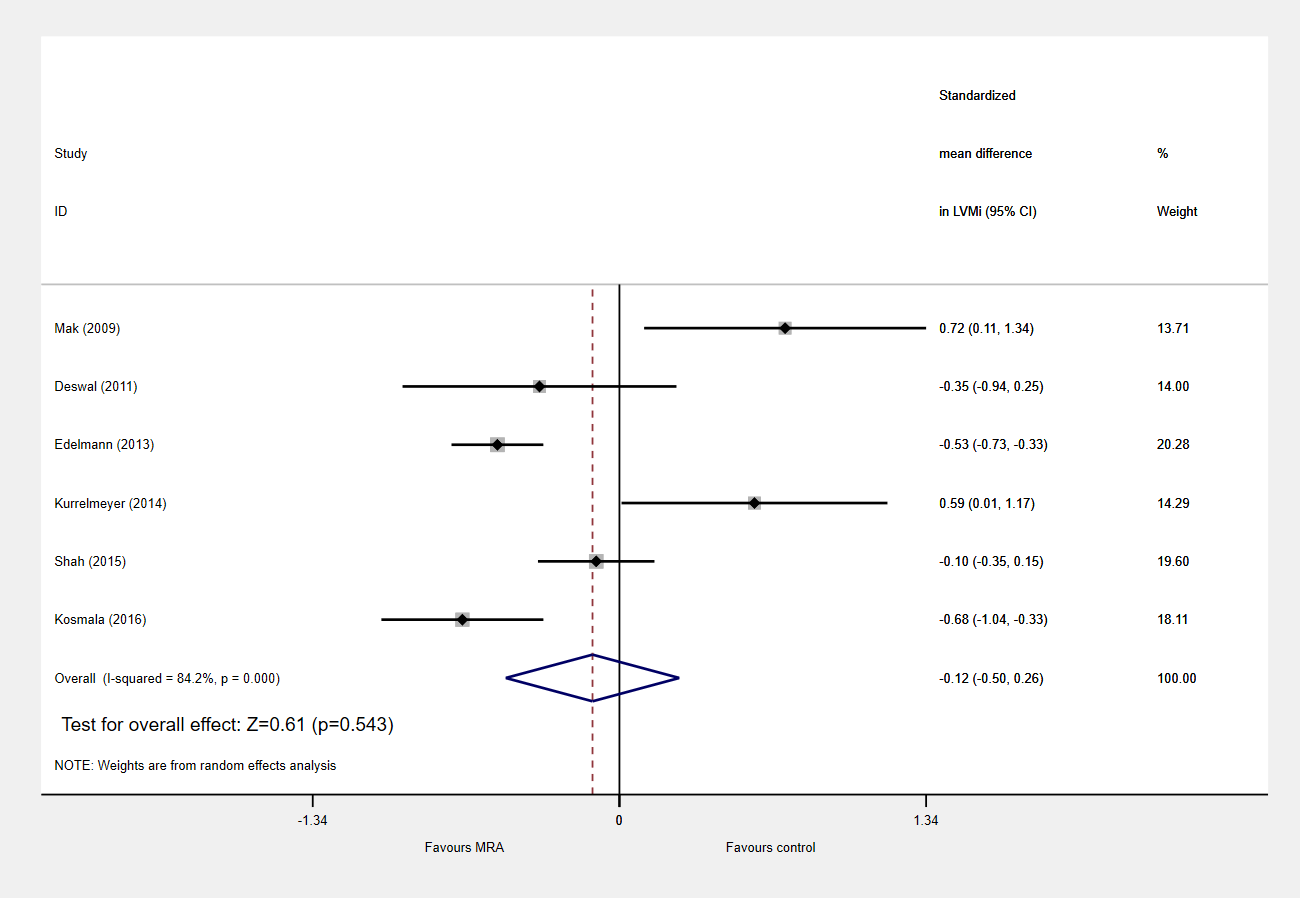


**Figure 1. Mean difference estimate of (a) deceleration time (DT), (B) left ventricular ejection fraction (LVEF) and (C) left ventricular mass index (LVMi) of MRAs versus control.**


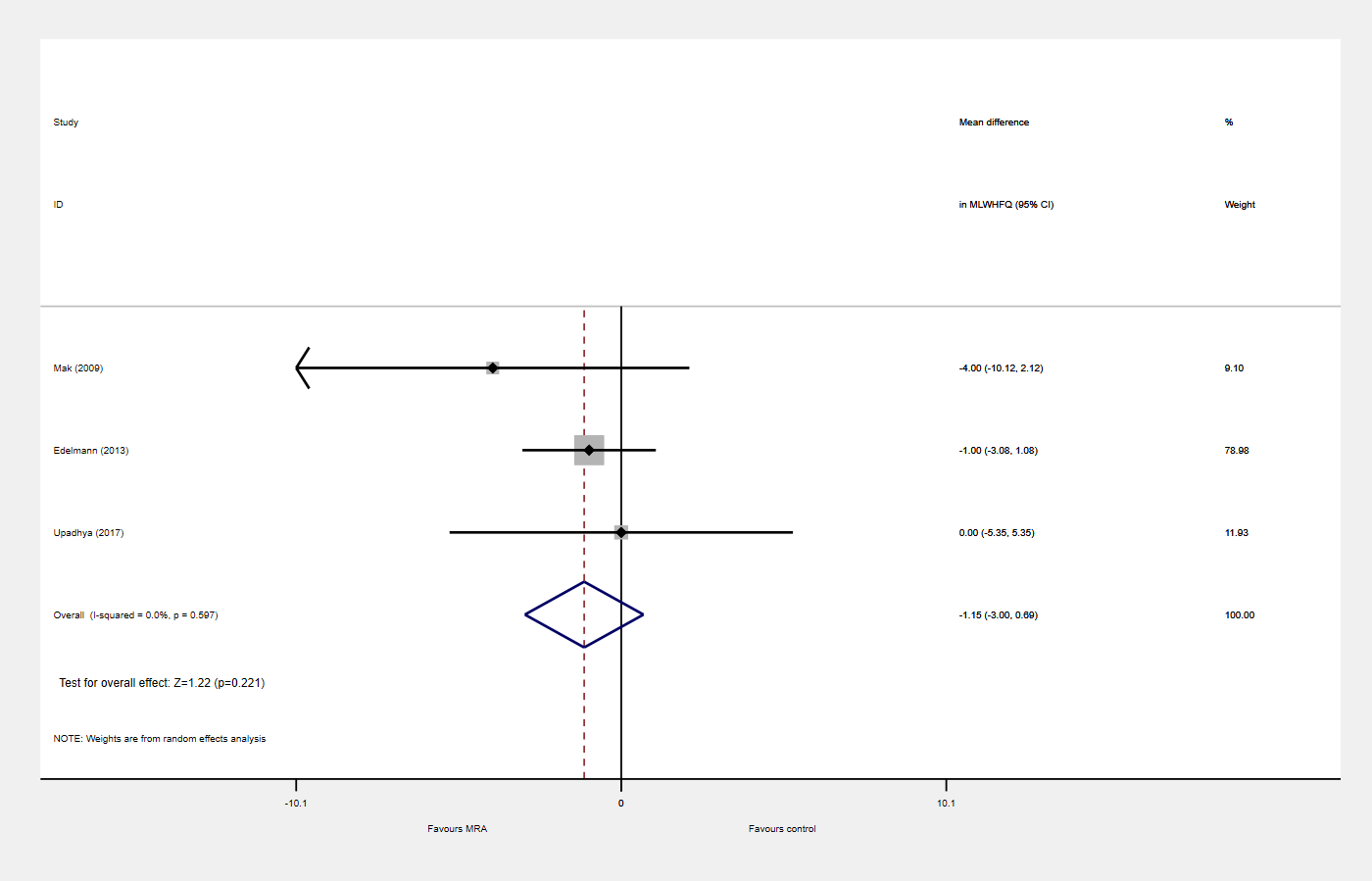


**Figure 2. Mean difference estimate of Milwaukee Living With Heart Failure Questionnaire (MLWHFQ) of MRAs versus control.**


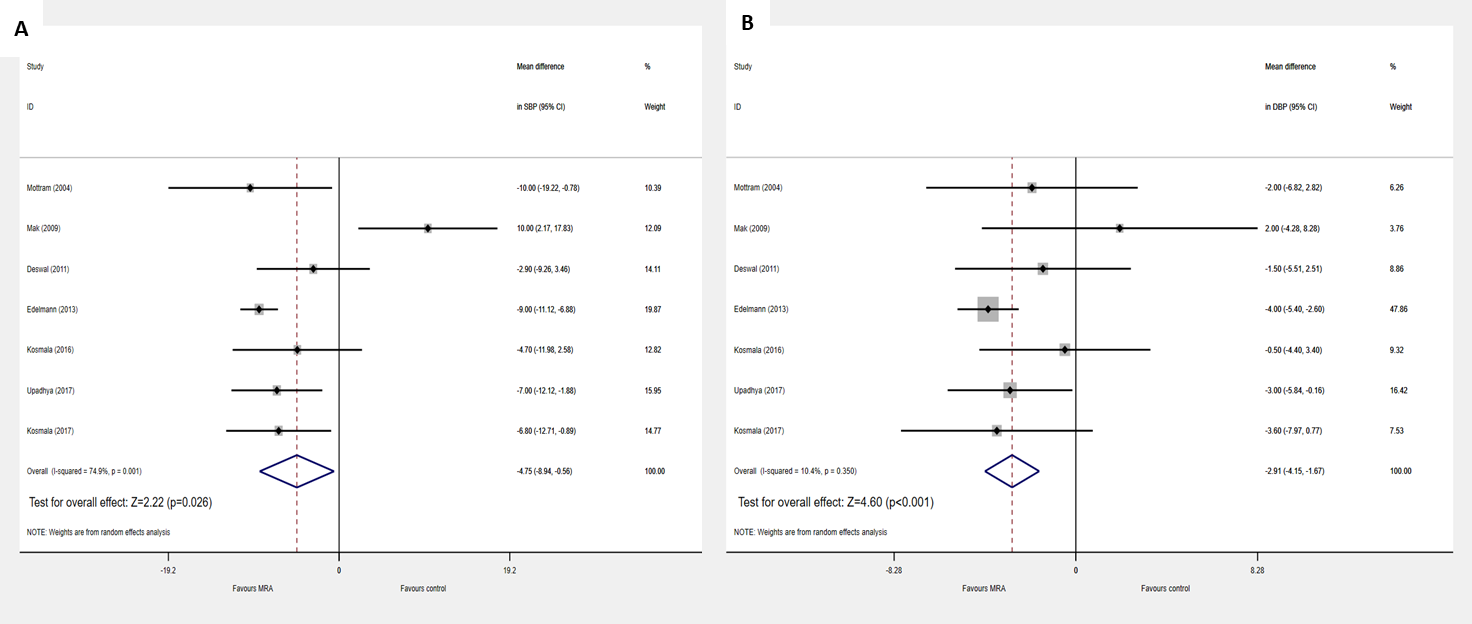


**Figure 3. Mean difference estimate of (a) systolic (SBP) and (B) diastolic blood pressure (SBP) of MRAs versus control.**


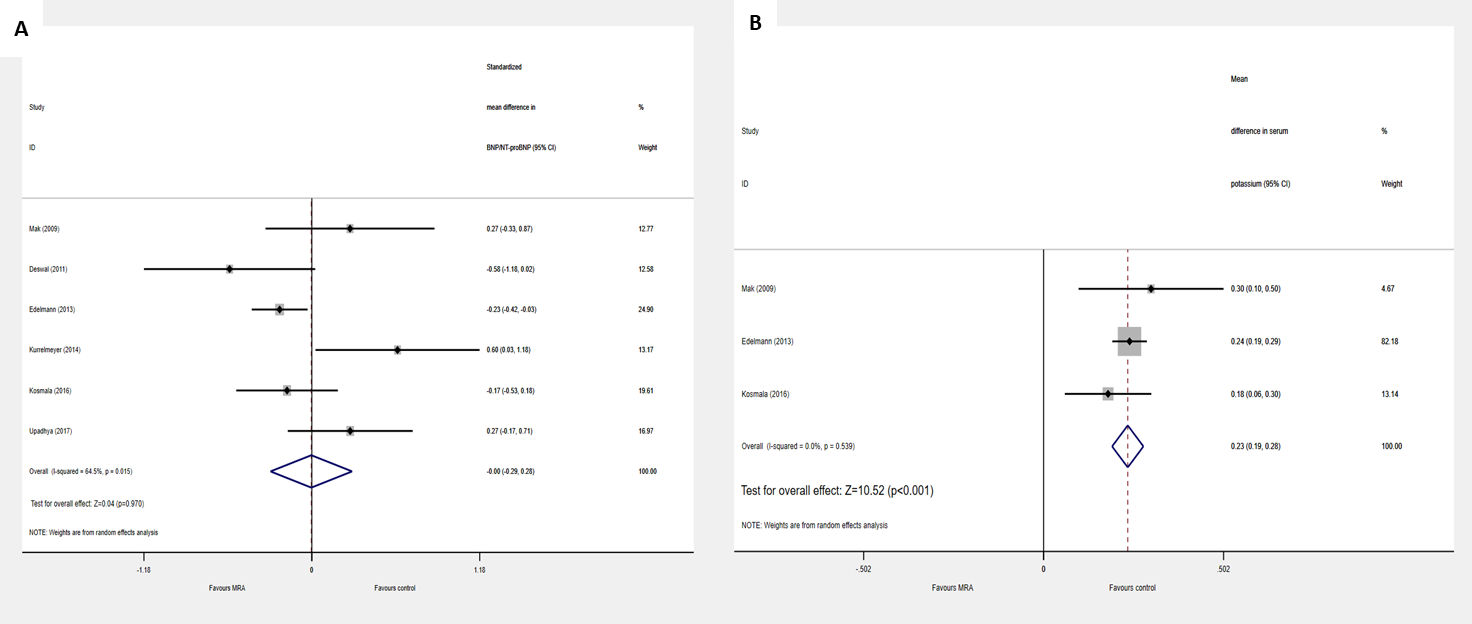


**Figure 4. Mean difference estimate of (a) BNP/NT-proBNP and (B) serum potassium of MRAs versus control.**
